# Insights from the degradation mechanism of cyclin D into targeted therapy of the cancer cell cycle

**DOI:** 10.1038/s41392-021-00732-y

**Published:** 2021-08-19

**Authors:** Maiwen Caudron-Herger, Sven Diederichs

**Affiliations:** 1 Division of RNA Biology & Cancer, German Cancer Research Center (DKFZ), Heidelberg, Germany; 2 Division of Cancer Research, Department of Thoracic Surgery, Medical Center—University of Freiburg, University of Freiburg, German Cancer Consortium (DKTK)—Partner Site Freiburg, Freiburg, Germany

**Keywords:** Oncogenes, Cell biology

The control of cell proliferation is an essential feature in development and tissue homeostasis and is prominently deregulated in tumors. Three publications in Nature now uncover the mechanism underlying the degradation of cyclin D^1–3^ and provide important insights for the targeted therapy of the cancer cell cycle.

Progression through the cell cycle is strictly governed by the consecutive activation of heterodimers of cyclins and cyclin-dependent kinases (CDKs). For the temporal control of the cell cycle from the G1 phase to mitosis, not only the tight regulation of the synthesis of the regulatory cyclin subunit is essential but also its timely degradation.

Cyclin D in complex with CDK4 or CDK6 drives progression from the G0 or G1 into the S phase and hence governs the important initiation of the cell cycle determining the cell for proliferation. Given its central role in cell division control, it is not surprising that the cyclin D-CDK4/6 complex is often hyperactivated in many cancer entities, e.g., by gene amplification or point mutations, and drives oncogenic cell proliferation.^4,5^ Nonetheless, the mechanism of cyclin D degradation remained a matter of debate.

Recently, multiple inhibitors for CDK4/6 such as palbociclib, abemaciclib, or ribociclib have been developed to suppress cyclin D-CDK4/6 driven proliferation of cancer cells. Indeed, CDK4/6 inhibitors are approved for the therapy of breast cancer and numerous studies are ongoing in other tumor entities.^4,5^ However, not all patients benefit from this therapeutic approach so that an intense search for predictive biomarkers as well as for mechanisms of primary or acquired resistance is required.^6,7^

A series of three publications have now uncovered the molecular mechanism of cyclin D destruction.^1–3^

The studies started from different scientific questions and approaches, but converged on the same findings. Simoneschi et al. searched for the ubiquitin ligase targeting D-type cyclins and employed three different approaches: a targeted siRNA screen for substrate receptors of CRL4 (DCAFS: DDB1-CUL4-associated factors) and a genome-wide CRISPR-Cas9 screen identifying factors impacting cyclin D abundance as well as a proteomic screen for cyclin D interactors.^1^ Chaikovsky et al. searched for modulators of the response to the CDK4/6 inhibitor palbociclib and employed a genome-wide CRISPR-Cas9 screen for factors altering cellular sensitivity.^2^ Maiani et al. searched for the mechanism of the impact of AMBRA1 (activating molecule in beclin-1-regulated autophagy) on cell proliferation and tumorigenesis.^3^

Although starting from these different questions, all three studies identified the protein AMBRA1 as the central factor controlling cyclin D degradation via the ubiquitin-proteasome pathway. The E3 ligase adaptor AMBRA1 bound to phosphorylated D-type cyclins and formed an active ubiquitin ligase complex with CUL4A, CUL4B, RBX1, and DDB1 called CRL4^AMBRA1^ (CUL4-RING E3 ubiquitin ligase).^1–3^ CRL4^AMBRA1^ marked cyclin D for degradation (Fig. 1a)—consequently, AMBRA1 loss led to stabilization of cyclin D (Fig. 1b). At the cellular level, loss of AMBRA1 caused increased cell proliferation and cell cycle progression and led to replication stress, while its overexpression reduced cell proliferation.^3^ In development, loss of AMBRA1 was embryonically lethal with severe neural development defects from increased cell proliferation and apoptosis which could be attenuated by CDK4/6 inhibition.^1,3^ In regard to cancer, AMBRA1 showed properties of a tumor suppressor gene as its loss promoted KRAS-driven lung adenocarcinoma^2,3^ and lymphoma^1^ growth in mice, it displayed cancer-associated mutations,^1,3^ low expression in tumors, and its loss associated with adverse outcome.^1,3^ Notably, cyclin D hotspot mutations in cancer abrogated AMBRA1 binding, thereby further corroborating the importance of this axis in cancer cell proliferation.^1^

**Fig. 1:**
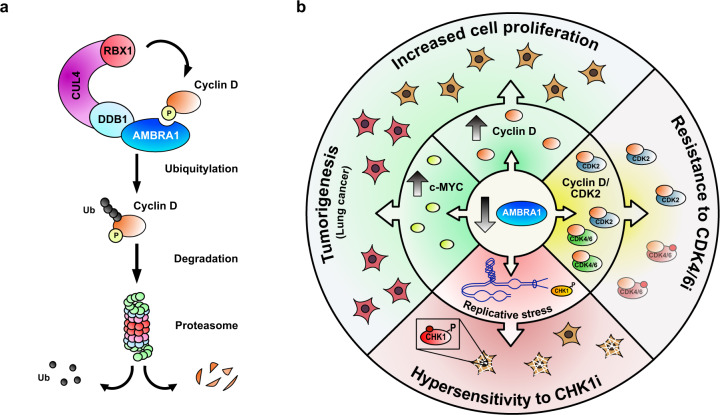
Impact of AMBRA1 depletion on cell proliferation and targeted therapy. **a** The CRL4^AMBRA1^ complex (E3 ligase complex including the proteins cullin4 (CUL4), RING box protein 1 (RBX1), damage-specific DNA binding protein 1 (DDB1) associated to AMBRA1) directly ubiquitylates (Ub chain) the phosphorylated form of cyclin D and targets it for degradation through the proteasome. **b** The loss of AMBRA1 results in the stabilization of cyclin D and members of the MYC protein family, which leads to increased cell proliferation and promotes tumorigenesis, as observed for lung adenocarcinoma and lymphoma, by executing its canonical role by activating the cell cycle kinases CDK4 and CDK6. In cells with a reduced amount of AMBRA1, cyclin D associates in addition with CDK2, so that cancer cells develop treatment resistance to CDK4/6 inhibitors, since the cyclin D–CDK2 complex is not sensitive to these inhibitors. In addition, AMBRA1-depleted cells face replicative stress due to the increased proliferation rate, which results in the activation of the checkpoint kinase CHK1 and the increased sensitivity to CHK1 inhibition.

For targeted therapy with CDK4/6 inhibitors, these studies are also of utmost importance: all three studies found a strong impact of AMBRA1 loss on the sensitivity of different cell types for the CDK4/6 inhibitors palbociclib or abemaciclib and thus could constitute a new mechanism of therapy resistance (Fig. 1b).^1,2,6,7^ Notably, AMBRA1 loss led not only to increased cyclin D levels but also promoted the formation of atypical cyclin D–CDK2 complexes. Since these active kinase complexes were insensitive to the inhibitors specific for CDK4/6, these could cause the resistant phenotype by bypassing CDK4/6 in the cell cycle.^1,2^ In turn, loss of CDK2 re-sensitized AMBRA1-deficient cancer cells to CDK4/6 inhibition^1^ emphasizing the need for combined CDK2/4/6 inhibitors (Fig. 1b).

In addition, AMBRA1 loss induced replication stress, which made the cells more sensitive to CHK1 inhibition (Fig. 1b).^3^ Next to CDK2, CHK1 may thus be an attractive drug target for inhibition by, e.g., AZD7762 to induce synthetic lethality for the treatment of AMBRA1-deficient tumors, which warrants and requires future pre-clinical and clinical validation to assess the usefulness of AMBRA1 as a predictive marker for therapy that determines the CDK4/6 inhibitor response of human tumors.

Overall, relevant research questions emanate from these findings especially regarding the linearity and causality of the proposed pathway: is AMBRA1 the only regulator of cyclin D degradation, or could parallel pathways affect its stability? In turn, does cyclin D mediate all functions of AMBRA1 in tumor suppression and CDK4/6 inhibitor resistance? The finding that cyclin D alone did not fully mimic the phenotype of AMBRA1 loss with respect to CDK4/6 inhibition may point toward additional changes beyond the mere induction of cyclin D,^2^ e.g., the cyclin D–CDK2 complexes or the induction of MYC family members.^3^ Lastly, the impact of AMBRA1 seems to depend on the genetic background: AMBRA1 correlates with survival in KRAS G12-mutant lung cancer, but not in KRAS wildtype or EGFR-driven lung cancer, which may reflect a more complex interplay of these pathways.^2^

In summary, these three publications thus uncover the molecular mechanism of cyclin D degradation via CRL4^AMBRA1^, establish AMBRA1 as a relevant tumor suppressor gene and sensitivity factor for CDK4/6 inhibition and provide hypotheses for therapeutic vulnerabilities of AMBRA1-deficient tumors by CDK2/4/6 or CHK1 inhibition with AMBRA1 loss also serving as predictive biomarker.^1–3^

Thereby, these studies in general provide prime examples of how basic research in molecular cancer biology, cell cycle, progression, and signal transduction can drive translational research and lead to important hypotheses for targeted therapy regarding predictive biomarkers and approaches to overcome resistance like the combination of CDK4/6 with CDK2 or CHK1 inhibition, which now need to be tested further towards the clinic.

## References

[1] Simoneschi D (2021). CRL4(AMBRA1) is a master regulator of D-type cyclins. Nature.

[2] Chaikovsky AC (2021). The AMBRA1 E3 ligase adaptor regulates the stability of cyclin D. Nature.

[3] Maiani E (2021). AMBRA1 regulates cyclin D to guard S-phase entry and genomic integrity. Nature.

[4] Otto T, Sicinski P (2017). Cell cycle proteins as promising targets in cancer therapy. Nat. Rev. Cancer.

[5] Sherr CJ, Beach D, Shapiro GI (2016). Targeting CDK4 and CDK6: from discovery to therapy. Cancer Discov..

[6] Herrera-Abreu MT (2016). Early adaptation and acquired resistance to CDK4/6 inhibition in estrogen receptor-positive breast cancer. Cancer Res..

[7] Wander SA (2020). The genomic landscape of intrinsic and acquired resistance to cyclin-dependent kinase 4/6 inhibitors in patients with hormone receptor-positive metastatic breast cancer. Cancer Discov..

